# A graph-based approach can improve keypoint detection of complex poses: a proof-of-concept on injury occurrences in alpine ski racing

**DOI:** 10.1038/s41598-023-47875-2

**Published:** 2023-12-05

**Authors:** Michael Zwölfer, Dieter Heinrich, Bastian Wandt, Helge Rhodin, Jörg Spörri, Werner Nachbauer

**Affiliations:** 1 Department of Sport Science, University of Innsbruck, 6020 Innsbruck, Austria; 2 Department of Electrical Engineering, Linköping University, Linköping, 581 83 Sweden; 3 Department of Computer Science, University of British Columbia, Vancouver, V6T 1Z4 Canada; 4 Department of Orthopaedics, Balgrist University Hospital, University of Zurich, 8006 Zurich, Switzerland

**Keywords:** Computer science, Scientific data

## Abstract

For most applications, 2D keypoint detection works well and offers a simple and fast tool to analyse human movements. However, there remain many situations where even the best state-of-the-art algorithms reach their limits and fail to detect human keypoints correctly. Such situations may occur especially when individual body parts are occluded, twisted, or when the whole person is flipped. Especially when analysing injuries in alpine ski racing, such twisted and rotated body positions occur frequently. To improve the detection of keypoints for this application, we developed a novel method that refines keypoint estimates by rotating the input videos. We select the best rotation for every frame with a graph-based global solver. Thereby, we improve keypoint detection of an arbitrary pose estimation algorithm, in particular for ‘hard’ keypoints. In the current proof-of-concept study, we show that our approach outperforms standard keypoint detection results in all categories and in all metrics, in injury-related out-of-balance and fall situations by a large margin as well as previous methods, in performance and robustness. The Injury Ski II dataset was made publicly available, aiming to facilitate the investigation of sports accidents based on computer vision in the future.

## Introduction

Deep learning-based keypoint detection defines the localization of anatomical landmarks or joints on the human body, such as shoulders, hips, and knees, which are detected and localized in an image or video to estimate the pose or configuration of a person. In sports, it is common to also consider non-anatomical landmarks (e.g., points on skis or other sports equipment) as keypoints. Since the emergence of deep convolutional neural networks (CNNs), such algorithms have become capable of automating manual digitization tasks impressively accurate^1,2^. They have become a standard method for a wide variety of motion capture applications, such as human computer interaction^3^, human activity recognition and video surveillance^4,5^, virtual and augmented reality^6,7^, or applications in sports science^8,9^ and health^10,11^.

Especially in the field of sports science and biomechanical research, deep learning-based methods offer possibilities that are difficult to realize with conventional motion capture systems. Beyond fast and efficient performance analysis and motion correction^9,11–14^, it might also allow for the reconstruction of sports accidents and the collection of kinematic data of the injury event^15,16^. Manual reconstruction of such accidents is restricted in objectivity, very time-consuming and thus limited to a small number of short video sequences^17^. State-of-the-art keypoint detection algorithms, in contrast, can detect multiple people simultaneously in real time with impressive accuracy^1,2,18,19^. In particular, this is true for common movements, which are represented in standard motion capture datasets. However, in regard to the detection of complex scenarios, such as injury situations in alpine ski racing, even the best performing algorithms struggle^1,15,16,20,21^. While state-of-the-art keypoint detectors were shown to perform very well in regular skiing situations, their performance drops sharply in fall situations^15,16^. An analysis of misdetected images revealed that occlusions due to snow spray and unusual poses were primarily responsible for the algorithm failures^16^. In such poses, limbs can be twisted, crossed and sometimes even the whole skier is upside down or in a horizontal position^15,16^. Similarly, difficulties with the detection of upside down or horizontal positions have been reported by many studies^1,18,20,21^. Based on this observation, a postprocessing tool that improves keypoint detection in fall situations was developed^15^. However, the performance of the proposed method was strongly dependent on the keypoint detection algorithm used, and for normal, upright poses, a slight decrease in performance was observed.

Therefore, we propose a new pre- and postprocessing routine to improve keypoint detection in injury scenarios. Similar to^15^, we leverage information from rotated image sequences to estimate a distribution of keypoints for each joint. While in^15^ a kinematic model was used to find the best keypoint among those keypoint estimates, we propose to select the best keypoints for every frame by formulating a shortest path problem to find an optimized path through the keypoint distribution. In addition, we extend the Injury Ski I^15^ dataset with 930 newly annotated injury images and evaluate our method on this new Injury Ski II dataset. Furthermore, we compare the performance of the keypoint detectors DCPose^1^ and AlphaPose^2^ in regular skiing, as well as in out-of-balance and fall scenarios, with and without our new method and with the kinematic model in^15^. Therefore, we evaluate the keypoint detector performance using the common metrics mean per joint position error (MPJPE), percentage of correct keypoints (PCK) and average precision (AP). With our method, the detection of correct keypoints in fall situations was increased by 16 and 22%, respectively, while the high performance in regular skiing situations could be maintained. The Injury Ski II dataset will be made available online for further research upon acceptance of this article.

## Related work

Human pose estimation has been a very active research field for many years. While in the beginning attempts were made to solve the problem by classical approaches using tree-structured or graphical model approaches^22–28^, great improvements have been achieved especially since the use of convolutional neural networks (CNNs)^2,18,29–34^. In particular, open source keypoint detection algorithms, such as OpenPose^18^, Detectron^19^ and AlphaPose^2^, have become increasingly popular in recent years due to their easy application in real-life situations. Most algorithms follow a top-down approach, in which first an object detector is employed to detect all humans and generate their respective bounding boxes before a pose estimator is applied on the cropped image of each detection^2,19,35,36^. While these approaches can benefit from very well-developed single pose estimation techniques, their performance is highly dependent on the particular person detector. Multiple person settings, occlusions or very unusual poses often lead to false person detection, making meaningful pose estimation difficult. In contrast, bottom-up approaches perform pose estimation on the entire image first, before the detected segments are then assigned to the respective individuals, e.g., using part affinity fields (PAFs)^18,32,37,38^. However, these approaches usually come at a much higher computational cost and were shown to be less accurate than state-of-the-art top-down methods.

Despite the many advances in human pose estimation, there are still many cases where even state-of-the-art algorithms fail to correctly identify all keypoints^16,18^. These include occluded or invisible keypoints, crowded background and highly flexed and/or extended and rotated poses^16,20,21^. Several research projects have focussed on locating such difficult keypoints^1,2,15,20,21^. By introducing a separate network branch that eliminates redundant person detections, AlphaPose achieved remarkably good results, both on current benchmark datasets and in highly complex scenarios, such as injury situations in alpine skiing^2,16^. Other approaches tackle such ‘hard’ keypoints by refining the keypoint estimates directly, either as part of their pipeline^21^ or as a postprocessing step that can be applied on top of any given keypoint detection algorithm^15,20^. Furthermore, as most keypoint detection algorithms only focus on upright poses with only small rotational changes in their training routines, they are not robust to largely rotated segments or poses^15,18,20^. By creating a path for learning new rotational changes based on a self-supervised method and combining them with the results obtained by a supervised model, great improvements for highly rotated poses of up to 15% were achieved^20^. Based on the same observation^15^, developed a postprocessing routine, which was shown to increase keypoint detection results in fall situations up to 21%. Similar to the method proposed in this article, input videos were rotated incrementally and keypoints detection was performed for every rotation. Thereby, a set keypoint estimates for every joint in every frame was obtained. In the following, such a set of keypoint estimates is defined as “keypoint candidates”, as only the best keypoints of those candidates are selected by the model. Over all frames, the keypoint candidates result in a keypoint distribution for each individual body joint. In^15^, the selection of the best keypoints among all keypoint candidates was then realized by a kinematic model, implemented as an alpha-beta-gamma filter. In this article, we propose to solve this task by formulating a shortest path optimization to find an ideal path through the keypoint distribution.

Deep-learning-based human pose estimation was first applied to alpine ski racing by Rhodin et al.^39^. They were able to reconstruct a skier’s 3D pose from a single perspective using a semisupervised multiview training approach. Based on this work, Ostrek et al.^40^ showed that the performance of deep learning-based approaches is accurate enough to address biomechanical research in this field. Subsequently, Bachmann et al.^41^ proposed a 3D bundle adjustment method to reconstruct a skier’s relative pose as well as its global position in a multicamera setting. While all these research projects focused on 3D pose estimation, only within the scope of the work of^41^, a 2D keypoint detector was specifically trained on alpine ski racing images using their ski-specific Ski 2D Pose dataset. However, this skiing specific dataset is small and shows limited variation, which in turn limits generalization, especially regarding injury analysis. Keypoint detection is a crucial part of 3D position estimation, since in most approaches, only the detected keypoints are lifted into 3D space. Thus, the pipeline’s overall performance is greatly dependent on the 2D keypoint detection results. Therefore, the performance of state-of-the-art 2D keypoint detectors in regular skiing situations as well as injury-related out-of-balance and fall situations were compared in a previous work^16^, and a rotational method to improve keypoint detection in such out-of-balance and fall situations was proposed^15^. Here, we present a further refinement of this method, which allows even more stable results in injury and fall situations.

## Dataset—Injury Ski II

Datasets containing the outdoor motion of any sport are highly desired and rare at the same time. Likewise, also publicly available datasets that cover sufficiently many images of skiers or ski racers are very sparse. Currently, there are only three such ski-specific datasets that are relevant for human pose estimation applications^15,41,42^. The Ski 2DPose dataset by Bachmann et al.^41^ contains approximately 2k images of different skiers in all disciplines, varying weather conditions and camera positions, as well as their respective 2D poses. Spörri et al.^42^ created the Ski-Pose PTZ-Camera Dataset by manually labelling approximately 20k images and calculating their respective 3D poses using passpoints and the method of direct linear transformation (DLT). Both datasets show only regular skiing situations and are therefore limited for use in injury analysis. For this reason, the Injury Ski I dataset was created^15^. This small dataset includes 533 images of skiers in all disciplines shortly before and during a fall that led to an injury. To expand this dataset, we create a new Injury Ski II Dataset by annotating an additional 930 images from nine different injury recordings. The recordings were obtained by television broadcasters or trainers and show world-cup athletes facing a fall, that subsequently led to an injury As in elite ski racing, the knee is the most frequently injured body part^43^, all nine videos covered by this dataset are knee injuries. The videos include five male and four female world-cup level ski racers in all disciplines (2x slalom, 2x giant slalom, 1x super-G, 4x downhill). In selecting the videos, care was taken to represent different video qualities, skier sizes and camera positions, only discarding very low-quality videos that could not be annotated in a meaningful way. Depending on the frame rate and video length, approximately 100 frames per recording were sampled and digitized using a custom-built LabVIEW script. Following^15,41^, 24 keypoints (14 body joints, 8 ski keypoints and 2 pole keypoints) were annotated per frame. Keypoints that were not visible were marked with − 1. To further promote research in the domain of computer vision and sports science, this injury-specific dataset is available at the following link: https://sport1.uibk.ac.at/mz/cv.

**Figure 1:**
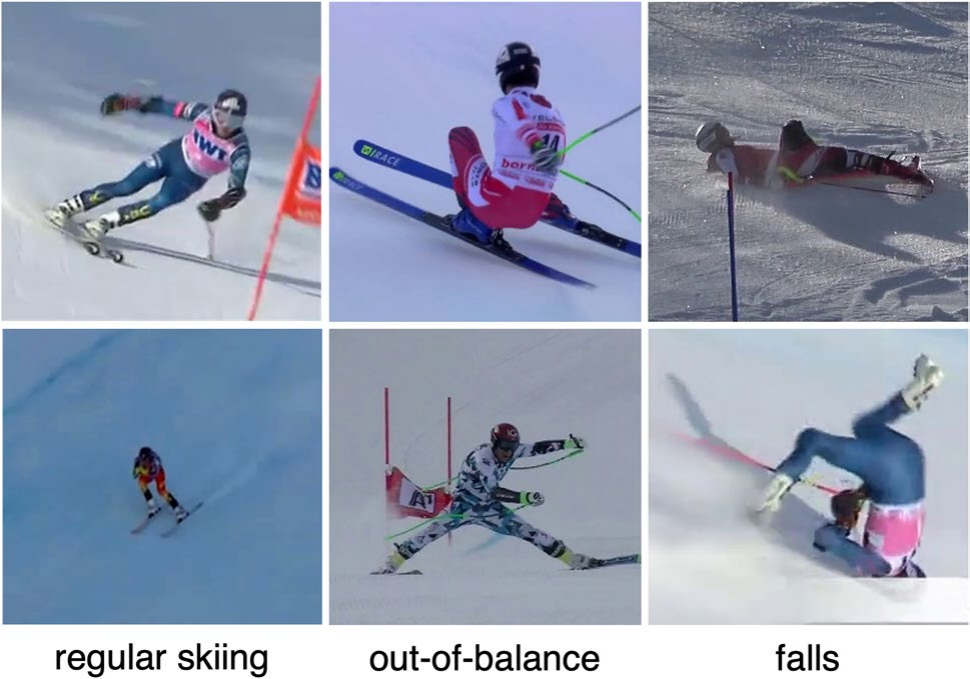
Example images of our ski- and injury-specific Injury Ski II dataset. Categorization of images regarding regular skiing, out-of-balance and fall situations.

## Methods

**Figure 2:**
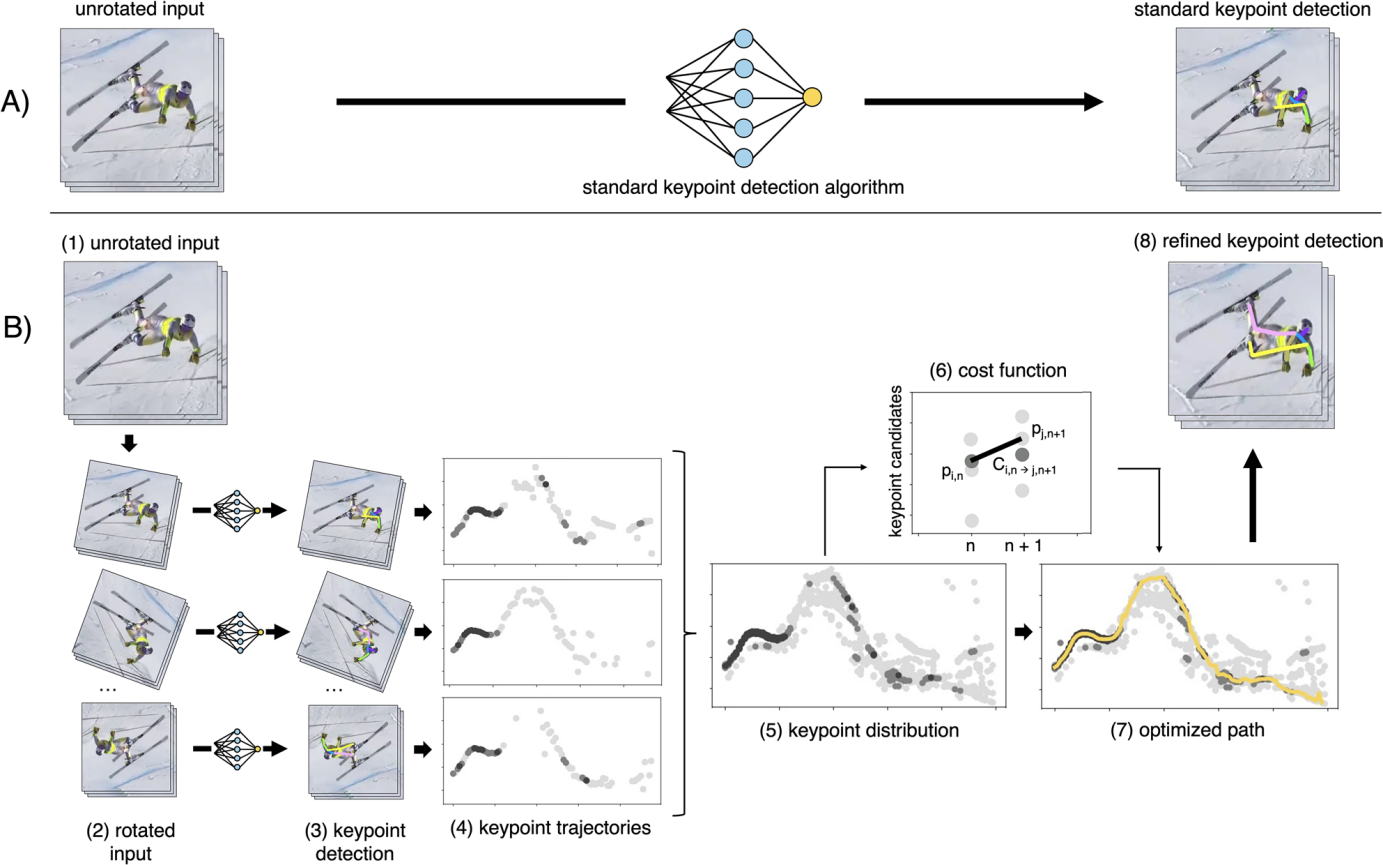
Schematic representation of our method (**B**) compared to standard keypoint detection (**A**). First, the input videos (1) are rotated incrementally in steps of $10^{\circ }$ between $0^{\circ }$ and $360^{\circ }$ (2). Afterwards, the keypoints are estimated by a keypoint detection algorithm, before being rotated back to their original orientation (3). This gives us 36 keypoint trajectories for each keypoint (4). Darker colored markers indicate keypoint candidates with a higher confidence value *c*. The trajectories of all rotations are then combined to one keypoint distribution per keypoint (5). Each keypoint candidate $p_{i,n}$ in a given frame *n* is linked to every keypoint candidate $p_{j,n+1}$ in the next frame $n+1$ by a cost $C_{i,n \rightarrow j,n+1}$ (6). Using Dijkstra’s shortest path optimization^44^, the ideal path through each keypoint distribution is determined (7). Finally, the refined pose is composed using the optimised keypoints only (8).

Within the scope of this proof-of-concept study, we developed a novel pre- and postprocessing routine that especially tackles ‘hard’ keypoints in injury-related out-of-balance and fall situations. The basic idea is based on the observation that skiers in injury situations can be rotated and individual limbs may be twisted. It has been shown that even state-of-the-art algorithms give incorrect pose estimates in such situations^16^. Since increasing random rotations in data augmentation at training the neural networks was reported to lead to an overall decrease in performance^15,18^, a postprocessing routine was developed that improves the detection in fall situations after applying an arbitrary keypoint detection algorithm^15^. We were able to improve this work by finding a novel approach that improves pose estimation in fall situations while being more robust to different keypoint detection algorithms and maintaining a high keypoint detection accuracy in regular skiing situations.

Following^15^, each input video was rotated incrementally from $0^{\circ }$ to $360^{\circ }$ in steps of $10^{\circ }$ and processed by a keypoint detection algorithm (Fig. 2). Since it was shown that DCPose and AlphaPose provide the best results in injury-related alpine skiing situations^16^, only these two algorithms are compared in this work. Predicted keypoints are then rotated back to their original orientation. Thereby, for each keypoint $p^k$, with $k = 1,...,K$, 36 keypoint candidates $p^k_{i,n} = (x^k_{i,n}, y^k_{i,n}, c^k_{i,n})$, with $i=1,...,36$, in each frame *n* are obtained. *K* is the number of keypoints per pose and can therefore vary depending on the model used. For simplicity, we drop the index *k*, and let $x_{i,n}$ and $y_{i,n}$ refer to the x and y coordinate and $c_{i,n} \in [0, 1]$ to the confidence value for each keypoint provided by the detection algorithm. We formulate the selection of the best keypoint candidate in each frame as the problem of finding the shortest path through a graph^44^. A graph is set up, connecting each keypoint candidate $p_{i,n}$ in frame *n* to every keypoint candidate $p_{j,n+1}$ in the next frame $n+1$ and assigning a cost $C_{i,n \rightarrow j,n+1}$ to each of these connections. The cost to get from $p_{i,n}$ to $p_{j,n+1}$ is composed of three parts: $C_C$, $C_D$ and $C_R$. The confidence cost $C_C$ is assigned as the inverse confidence value of the incoming keypoint candidate $c_{i,n}$, the distance cost $C_D$ is calculated as the Euclidean distance between $p_{i,n}$ and $p_{j,n+1}$ and the rotation cost $C_R$ penalizes large differences in the rotation angles $r_{i,n}$ and $r_{j,n+1}$ of the respective keypoint candidates. All contributions are multiplied by the respective coefficients *c*, *d* and *r*, as shown in equation 1.

1 $$\begin{aligned} \begin{gathered} C_{i,n \rightarrow j,n+1} = c \cdot C_{C} + d \cdot C_{D} + r \cdot C_{R}, \\ \text {with}\quad C_C = \frac{1}{c_{j,n+1}}, \quad C_D = \sqrt{(x_{i,n}-x_{j,n+1})^{2} + (y_{i,n}-y_{j,n+1})^{2}}, \quad C_R = \left| \sin \left( \frac{r_{i,n} - r_{j,n+1}}{2}\right) \right| . \end{gathered} \end{aligned}$$

We use Dijkstra’s shortest path algorithm^44^ to find the path with the lowest overall cost through the keypoint distribution for a given set of hyperparameters *c*, *d* and *r*. To make full use of all injury-specific data available, we merge the Injury Ski I and Injury Ski II datasets and split them into parts of eight videos each. Thus, we obtain a validation set containing 805 frames and a test set containing 658 frames. To optimize our hyperparameters *c*, *d* and *r*, we perform an exponential grid search on the validation set. The best overall performance is found for $c = 0.01$, $d = 1$ and $r = 0.01$.

Following^15^, we evaluate the performance of our method with respect to different skiing situations. Therefore, all frames of our test dataset are split into regular skiing, out-of-balance and fall situations (see Fig. 1). Regular skiing situations include all situations in which the skiers demonstrate controlled skiing. Out-of-balance situations include situations in which the skiers are out of control but still stand on their skis and try to regain balance. Finally, fall situations include all images in which skiers have already hit the ground or do not have contact with the ground at all. DCPose and AlphaPose are then run in their pretrained configuration on the test set, with and without applying our method. The results are evaluated using the average precision (AP), percentage of correct keypoints (PCK) and the mean per joint position error (MPJPE) metrics. The PCK threshold is chosen to be 20% of the torso diameter (PCK@0.2).

**Figure 3:**
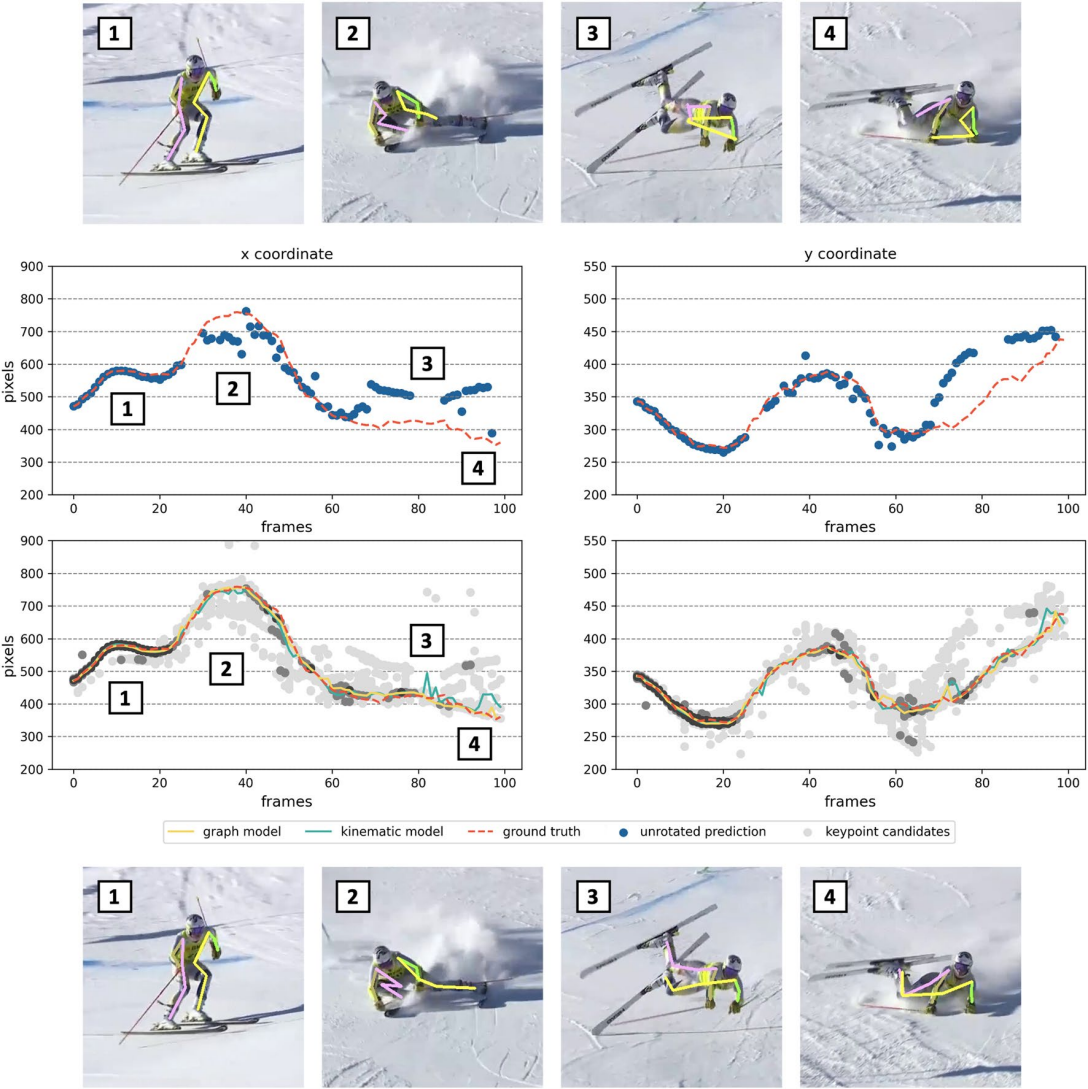
Example of the left ankle coordinates *x* and *y* of video number 2 detected by DCPose. The first row shows reference images overlaid with unrotated DCPose detections. The same images overlaid with our refined keypoint estimates are presented at the bottom for comparison. The first charts of the middle section show the ground truth ankle trajectory in red as well as the unrotated standard DCPose detections in blue. Gaps in the sequence of unrotated detections indicate frames where the corresponding keypoint was not detected. Below you find the keypoint distribution of all rotations. A darker shading indicates a higher confidence value. The ground truth is again shown in red, our refined model in orange and the kinematic method^15^ in blue.

**Figure 4:**
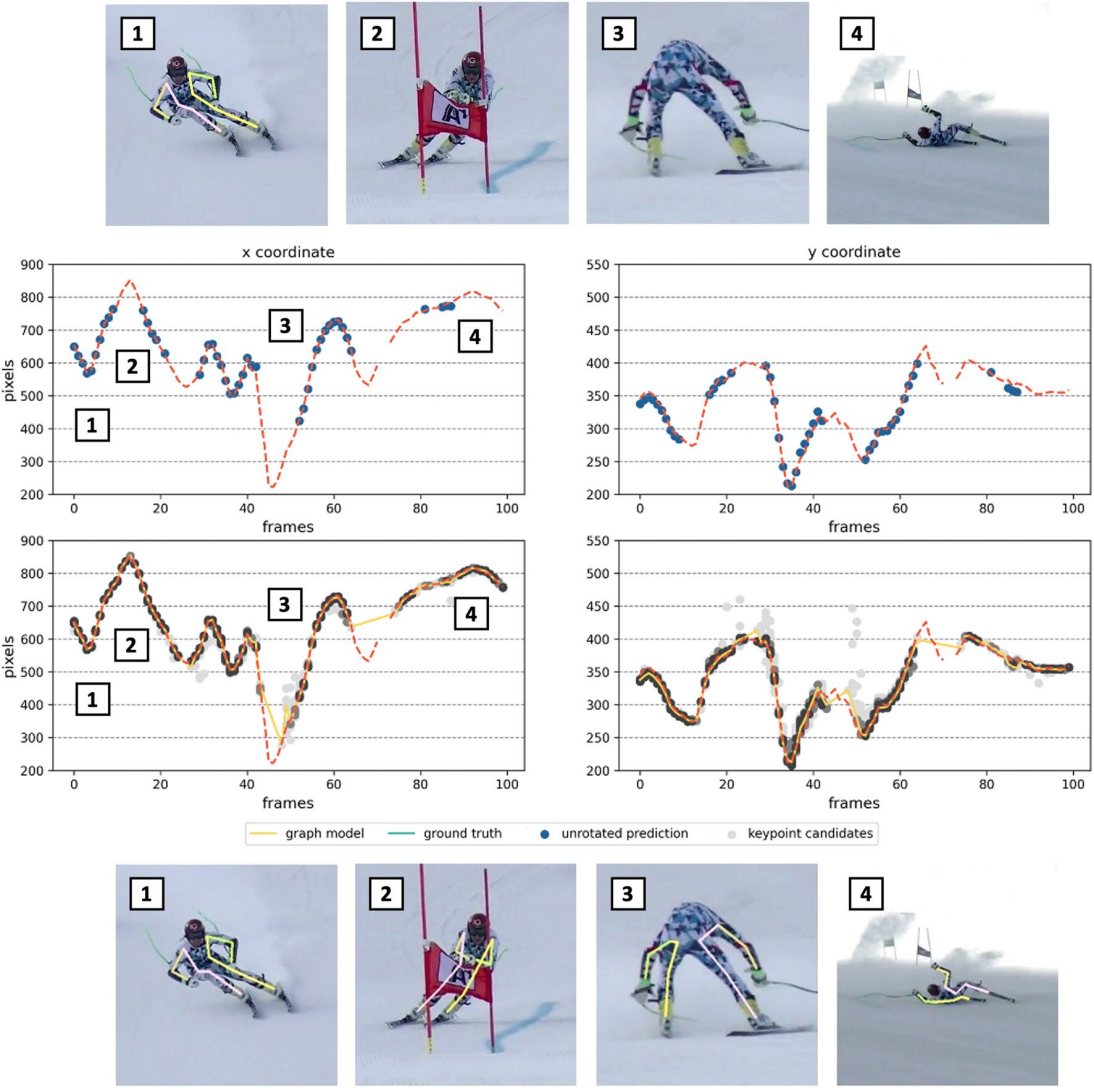
Example of the head coordinates *x* and *y* of video number 12 detected by DCPose. The first row shows reference images overlaid with unrotated DCPose detections. The same images overlaid with our refined keypoint estimates are presented at the bottom for comparison. The first charts of the middle section show the ground truth head trajectory in red as well as the unrotated standard DCPose detections in blue. Gaps in the sequence of unrotated detections indicate frames where the corresponding keypoint was not detected. Below you find the keypoint distribution of all rotations. A darker shading indicates a higher confidence value. The ground truth is again shown in red, our refined model in orange and the kinematic method^15^ in blue.

## Results

**Table 1:** Comparison of both algorithms without any pre- and postprocessing vs. the kinematic method^15^ vs. our results by the mean per joint position error, percentage of correct keypoints (PCK) and average precision (AP) metric with respect to regular skiing (reg), out-of-balance (oob) and fall frames.

	MPJPE				PCK				AP			
	All	reg	oob	Fall	All	reg	oob	Fall	All	reg	oob	Fall
DCPose	16.4	9.5	17.3	30.5	0.75	0.87	0.84	0.43	0.55	0.71	0.65	0.13
DCPose + kinematic^15^	13.7	9.4	15.8	18.6	0.83	0.89	0.87	0.66	0.61	0.70	0.69	0.33
DCPose + ours	13.3	9.1	14.4	20.9	0.84	0.90	0.90	0.65	0.65	0.73	0.73	0.35
AlphaPose	15.0	8.5	17.8	24.6	0.83	0.92	0.88	0.58	0.61	0.77	0.66	0.27
AlphaPose + kinematic^15^	14.6	9.6	17.7	19.2	0.83	0.91	0.86	0.65	0.59	0.70	0.64	0.33
AlphaPose + ours	12.5	8.3	15.6	15.6	0.86	0.92	0.90	0.72	0.65	0.77	0.71	0.44

Without applying our method, we observed a high performance for algorithms in regular skiing situations. Specifically, 9 out of 10 keypoints were estimated correctly at MPJPEs of less than 10 pixels and an average precision above 0.71 (see Table 1). Towards out-of-balance situations, we observed a slight decrease in all metrics for both algorithms. The PCK decreased by $3\%$ for DCPose and $4\%$ for AlphaPose, while MPJPEs roughly doubled. In AP, the decline was more pronounced, with 0.06 and 0.11, respectively. In fall situations, the performance of both algorithms dropped sharply in all metrics. PCK sank to $43\%$ (DCPose) and $58\%$ (AlphaPose), while AP dropped to 0.13 and 0.27, respectively. MPJPEs became three times as high as in regular skiing situations. Across all categories and metrics, AlphaPose outperformed DCPose in this comparison. AlphaPose achieved an $8 \%$ higher average PCK, with a 1.4 pixel lower mean error and a 0.06 higher AP. The superiority of AlphaPose became especially clear in fall situations. Here, AlphaPose was ahead by $15\%$ in PCK, 0.14 in AP and by 5.9 pixels in MPJPE.

Our new pre- and postprocessing routine significantly improved the performance of both algorithms. Benefits were observed across all categories as well as in all metrics. Even in regular skiing situations, when skiers are merely upright and already well detected by standard keypoint detection algorithms, PCK was slightly improved by up to $3\%$, and increases of $6\%$ (DCPose) and $2\%$ (AlphaPose) were observed in out-of-balance situations. The largest improvements were made in fall situations with a plus of $22\%$ and $14\%$ in PCK, respectively. A very similar pattern was found in AP. In regular skiing situations, high performance was maintained and/or slightly improved by up to 0.02. However, in out-of-balance situations, we observed increases of 0.08 for DCPose and 0.11 for AlphaPose, and in fall situations, the results even improved by 0.22 and 0.17, respectively. Accordingly, the MPJPE metric also showed a improvement of up to 0.4 pixels for regular skiing, while in out-of-balance situations the errors decreased significantly by 2.9 and 2.2 pixels, respectively. In fall situations, reductions of 9.6 and 9.0 pixels, which correspond to $31 \%$ and $37 \%$, respectively, were observed.

We also run^15^ on this dataset. For DCPose, improvements on^15^ in comparison to baseline (standard detections) were observed in out-of-balance and especially in fall situations in all metrics. In regular skiing situations, the improvements in PCK and MPJPE were marginal, while a slight decrease in AP was found. For Alphapose, however, only fall situations were improved compared to baseline in all metrics, while in regular skiing and out-of-balance situations, a decline was observed in PCK and AP (Table 1). By contrast, our new method consistently improves standard keypoint detection in all categories and metrics.

Figures 3 and 4 show example keypoint distributions for the DCPose algorithm. In the top and bottom row, example images overlayed with the standard prediction (top) and the same images overlayed with the refined keypoint estimates (bottom) are presented. In the middle part, example keypoint distributions are shown. In the first graphs, the unrotated predictions in blue and the ground truth in red are plotted. The second row shows the distribution of all 36 keypoint candidates, ground truth and our refined model in yellow. A darker shading of the keypoint candidates indicates a high confidence value.

## Discussion

The main contributions of this work were as follows: (1) We proposed a novel graph-based pre- and postprocessing routine to improve keypoint detection in ‘hard’ scenarios, such as out-of-balance and fall situations in alpine ski racing. (2) We showed that our method outperformed plain state-of-the-art 2D keypoint detection in all categories (i.e., regular skiing, out-of-balance and falls) and metrics (i.e., PCK, AP and MPJPE). (3) We extended the Injury Ski Dataset, providing another 930 ski- and injury-related images and their corresponding 2D poses.

Rotating each input image, running keypoint detection and transforming the keypoint estimates back to their original orientation allowed us to generate multiple keypoint candidates for each keypoint in each frame (Figs. 3 and 4). Given the keypoint candidates of all frames, the selection of the best keypoint candidates was accomplished by formulating a shortest path optimization. In the underlying graph, the cost of getting from one keypoint candidate in a given frame to another keypoint candidate in the next frame, referred to as an edge in graph theory, was defined based on the following observations. (1) The confidence value of each keypoint estimate correlates with the observed quality of the estimation. Although sometimes high confidence keypoints may be wrong, they usually match the ground truth well. Therefore, a confidence cost $C_C$ was assigned as the inverse confidence value of the incoming keypoint candidate, penalizing connections to low confidence keypoint estimates. (2) Keypoint trajectories are smooth, rarely showing large gradients. By defining a distance cost $C_D$, keypoint candidates that lied close to each other were favoured. This ensured that the overall model stayed smooth and outliers, even if they had a high confidence value, were discarded. (3) Rotational changes from one frame to another are relatively small, and fast rotational changes are therefore unlikely. Penalizing such fast changes in the rotation angle between the respective keypoint candidates by a cost proportional to the angular distance $C_R$, the performance on our validation set was further increased. With this method, we provide an improved alternative to^15^. Both methods take the confidence value, the spatial distance and the angular distance between two keypoint candidates into account when determining the best keypoint candidate in each frame. However, in contrast to the graph-model-based approach proposed in this article, in^15^, the best keypoints were selected using a kinematic model based on an $\alpha$-$\beta$-$\gamma$ filter. Furthermore, validation and testing were performed on a three times smaller dataset.

Analysing the keypoint distributions revealed interesting findings about the keypoint detector performance, particularly at ‘hard’ out-of-balance and fall frames. Narrow keypoint distributions were found in regular skiing situations at sufficiently high image qualities. In these frames, the standard (unrotated) predictions align very well with all other keypoint candidates as well as the ground truth data (Fig. 3 (1)). The high performance of both keypoint detection algorithms, even without applying our method, is expressed by these narrow keypoint distributions. Towards out-of-balance and fall situations, keypoint estimates scatter, resulting in a wider and more diffuse keypoint distribution. While large deviations between the standard predictions and ground truth were frequently observed in these situations, estimates from other rotation angles could match the ground truth very well (Fig. 3 (2)–(4)). A cross-check with the corresponding videos revealed that these diffuse keypoint distributions correlate with twisted and compressed poses, often combined with partial occlusions due to snow spray (Fig. 3 (2)). These hard poses may occur due to high ground reaction forces when hitting the snow surface, e.g., after a fall, or when one ski suddenly catches an edge, after slipping away during a turn, as described by the slip-catch mechanism, the most frequent mechanism of ACL injuries in alpine ski racing^45^. In out-of-balance and fall situations, bimodal keypoint distributions were also observed. In these cases, crossed limbs and/or self-occlusion led to mismatched keypoints, as shown in Fig. 3 (4). Finally, there were also very thin distributions in which only very few keypoints were detected at all. These thin keypoint distributions, mainly caused by occlusions due to snow spray, external objects or the skier itself and/or a very low image quality, led to no keypoint detection in the unrotated input frame at all. In most of these cases, keypoint estimates were still found in other rotations. This shows that our approach not only improves largely rotated poses but also provides valuable information for ‘hard’ keypoints, such as occluded keypoints, in general (Fig. 4 (2)–(4)). Comparing both algorithms, AlphaPose provided generally narrower and denser keypoint distributions with a larger number of high confidence keypoints, which explains the overall superior performance of AlphaPose over DCPose. All these findings are very much in line with^15^ as well as the observations of^18,20^, who identified rotated poses as a major source of errors for standard keypoint detection algorithms. In all of the situations described above, additional information about the keypoint location is supplied by the keypoint distributions.

Without applying any pre- and postprocessing, we observed a high keypoint detection accuracy in regular skiing situations. The detection results decreased towards out-of-balance situations, while the performance dropped sharply in fall situations. This matched the results of^15,16^ and was in good agreement with the observations on the keypoint distributions discussed above. Large deviations between standard detection results and ground truth were predominantly observed in out-of-balance and fall situations, where keypoint distributions became diffuse or sparse. Furthermore, we also compared the DCPose and AlphaPose results to their original literature. DCPose reached 0.79 AP on the PoseTrack 2018 test set^1^, while AlphaPose achieved 0.73 AP on the COCO test set^2^. Despite the very different test data, our results on regular skiing situations of 0.71 (DCPose) and 0.77 (AlphaPose) in AP match with the literature quite well.

Our new method significantly improved the performance of both the DCPose and AlphaPose, across all categories and in all metrics. The effects were less pronounced in regular skiing situations than in out-of-balance situations. The largest improvements were achieved in fall situations. In regular skiing situations, PCK and AP were only marginally improved, while MPJPEs were reduced by up to $10 \%$. Maintaining the inherently high performance of both algorithms in this regime and even achieving delicate improvements can be considered a success of our method. The very sharp keypoint distributions observed in these situations indicate that major improvements are hardly possible. In contrast, in out-of-balance situations, large benefits were observed. With an improvement in PCK by up to 6% and 0.08 in AP, the detection results for both algorithms were elevated to the regular skiing level. Both exemplary keypoint distributions (Figs. 3 and 4) showed how our refined model may benefit from the rotated keypoint estimates to compensate for misdetected standard keypoint estimates. In fall situations, such ‘hard’ keypoints became more frequent. Therefore, keypoint detection results could profit even more from our method. We report improvements in PCK by $22\%$ for DCPose and $14\%$ for AlphaPose. Accordingly, AP was elevated by 0.22 and 0.17. Reductions in MPJPE of $31\%$ and $37\%$ were observed.

Our new method outperformed the kinematic model described by^15^ in just about all categories (i.e., regular skiing, out-of-balance and fall situations) and metrics. Only in the fall category for DCPose did both algorithms perform similarly, with a marginal advantage for^15^ in PCK and MPJPE. While the improvements in fall situations were large, e.g., $23\%$ for DCPose and $7\%$ for AlphaPose in PCK, in regular skiing and out-of-balance situations, improvements were only achieved for the DCPose algorithm. For AlphaPose, a decrease in both metrics (PCK and AP) was found. As mentioned above, differences between AlphaPose and DCPose were clearly reflected in their keypoint distributions. Whether our new method or the kinematic model works well depends on the information provided by these keypoint distributions. Therefore, our results lead us to conclude that the application of the graph-based model is more robust to different keypoint distributions and therefore to different keypoint detection algorithms than the kinematic model used before. The novel method proposed in this study improved the results regardless of the applied keypoint detection algorithms and can be applied to any input video.

To the best of our knowledge, there is only one other study that offers a refinement of keypoint detection for rotated settings as a postprocessing tool that can be applied to any keypoint detection algorithm^20^. In this approach, a semisupervised neural network is trained on learning new rotational changes and combining the results with conventional keypoint detection results. Similar to our results, the authors report standard keypoint detection results in the range of 0.7 and 0.8 in AP. For largely rotated images, AP dropped to below 0.1 and 0.3 on the COCO dataset, depending on the keypoint detection algorithm used. However, as this method was tested on different datasets, images were artificially rotated and other keypoint detection algorithms were used, a direct quantitative comparison of the methods improvement is not reasonable.

Finally, we discuss our extended Injury Ski dataset. Adding another 930 images of professional alpine ski racers in injury situations, we can now provide a total of 1466 ski- and injury-related images plus their respective 2D poses. This makes our dataset similar in size to the only other publicly available 2D dataset for alpine skiing^41^. This dataset covers regular skiing images sampled from 16 different video recordings showing mainly competitive ski racers from many different perspectives in various weather conditions. Similarly, our combined Injury Ski dataset covers 16 elite athletes in all alpine skiing disciplines (slalom, giant slalom, super-G and downhill) and varying image qualities. Since all images were annotated following the annotation scheme of^41^, the Ski2D Pose dataset and Injury Ski dataset can be merged easily to exploit an even more powerful dataset to develop computer vision applications for alpine skiing, in particular with a focus on ski accidents. Other than these two datasets, only one other skiing related dataset is publicly available. While this comparatively large 3D Ski-Pose PTZ-Camera dataset^42^ provides 3D motion capture data of approximately 20k labelled skier frames of six elite ski athletes, it only covers three subsequent turns of a single GS track.

## Limitations

This study is limited by the relatively small size of our dataset. Although we could triple the number of ski- and injury-specific frames compared to previous work^15^, more frames in each category would make our results more robust and would allow us to fine-tune deep learning methods. However, the availability of exploitable injury footage is limited, as accidents are seldom and can hardly be staged. Furthermore, only the two best performing keypoint detection algorithms of^16^ were investigated in this study. Concluding that our method works well for any given keypoint detection algorithm should be confirmed on a larger number of algorithms.

## Conclusion

Computer vision-based motion capture offers fantastic opportunities in sports science analysis. At the same time, the complex and often fast movements in some sports pose great challenges to human observers but equally to automated keypoint detection algorithms. For example, when analysing injuries in alpine ski racing, situations often arise in which individual limbs are occluded or twisted, or even the entire athlete is upside down. In this article, we propose a new method that leverages rotated input images to improve keypoint detection in such situations. The method can be applied as pre- and postprocessing independent of the chosen keypoint detection algorithm. We have shown that our approach outperforms standard keypoint detection results in all categories and in all metrics, especially for ‘hard’ keypoints. In fall situations, we observed a reduction in the mean per joint position error of approximately one-third, while the percentage of correct keypoints was increased by up to 22%. We were also able to outperform previous methods, such as^15^, in performance and robustness. We report that AlphaPose is superior to DCPose in all metrics and categories on our dataset. Furthermore, we extended the Injury Ski dataset and made it publicly available for further research.

## Data Availability

The datasets generated during and analysed during the current study are available in the ‘injuryski’ and ‘injuryskiII’ repositories, https://sport1.uibk.ac.at/mz/cv.

## References

[1] Liu, Z. *et al.* Deep dual consecutive network for human pose estimation. In *Proceedings of the IEEE/CVF Conference on Computer Vision and Pattern Recognition*, 525–534 (2021).

[2] Fang, H. S., Xie, S.,Tai, Y. W. & Lu, C. RMPE: Regional multi-person pose estimation. In *ICCV*, (2017).

[3] Salti, S., Schreer, O. & Di Stefano, L. Real-time 3d arm pose estimation from monocular video for enhanced HCI. In *Proceedings of the 1st ACM Workshop on Vision Networks for Behavior Analysis*, VNBA ’08, 1–8, 10.1145/1461893.1461895 (Association for Computing Machinery, 2008).

[4] Khurana, R. & Kushwaha, A. K. S. Deep learning approaches for human activity recognition in video surveillance—A survey. In *2018 First International Conference on Secure Cyber Computing and Communication (ICSCCC)*, 542–544, 10.1109/ICSCCC.2018.8703295 (2018).

[5] Sreenu G, Durai S (2019). Intelligent video surveillance: A review through deep learning techniques for crowd analysis. J. Big Data.

[6] Liu, X., Feng, X., Pan, S., Peng, J. & Zhao, X. Skeleton tracking based on kinect camera and the application in virtual reality system. In *Proceedings of the 4th International Conference on Virtual Reality*, ICVR 2018, 21-25, 10.1145/3198910.3198915 (Association for Computing Machinery, 2018).

[7] Ro, H., Park, Y. J., Byun, J.-H. & Han, T.-D. Display methods of projection augmented reality based on deep learning pose estimation. In ACM SIGGRAPH,. Posters. *SIGGRAPH ’* **19**, 2019. 10.1145/3306214.3338608(Association for Computing Machinery, New York, NY, USA

[8] Cust E, Sweeting A, Ball K, Robertson S (2019). Machine and deep learning for sport-specific movement recognition: A systematic review of model development and performance. J. Sports Sci..

[9] Pandurevic D, Draga P, Sutor A, Hochradel K (2022). Analysis of competition and training videos of speed climbing athletes using feature and human body keypoint detection algorithms. Sensors.

[10] Shapoval S, Zapirain B, Zorrilla A, Mugueta-Aguinaga I (2021). Biofeedback applied to interactive serious games to monitor frailty in an elderly population. Appl. Sci..

[11] Lonini L (2022). Video-based pose estimation for gait analysis in stroke survivors during clinical assessments: A proof-of-concept study. Digit. Biomark..

[12] Pandurevic D, Draga P, Sutor A, Hochradel K (2022). Analysis of competition and training videos of speed climbing athletes using feature and human body keypoint detection algorithms. Sensors.

[13] Chen, S. & Yang, R. R. Pose trainer: correcting exercise posture using pose estimation. arXiv preprint arXiv:2006.11718 (2020).

[14] Wang, J., Qiu, K., Peng, H., Fu, J. & Zhu, J. AI coach: Deep human pose estimation and analysis for personalized athletic training assistance. In *Proceedings of the 27th ACM International Conference on Multimedia*, 374–382 (2019).

[15] Zwölfer, M. *et al.* Improved 2D keypoint detection in out-of-balance and fall situations—combining input rotations and a kinematic model, 10.48550/ARXIV.2112.12193 (2021).

[16] Zwölfer, M. *et al.* Deep learning based 2D keypoint detection in alpine skiing—a performance analysis of state-of-the-art algorithms. In *Book of Abstracts of the Joint Conference 24th International Congress on Snow Sport Trauma and Safety-37th Congress of the International Society for Snowsports Medicine.*, 60 (2022).

[17] Bere T (2013). Kinematics of anterior cruciate ligament ruptures in world cup alpine skiing: 2 case reports of the slip-catch mechanism. Am. J. Sports Med..

[18] Cao, Z., Hidalgo, G., Simon, T., Wei, S. E. & Sheikh, Y. Openpose: Realtime multi-person 2d pose estimation using part affinity fields (2019). 1812.08008. 10.1109/TPAMI.2019.2929257 31331883

[19] Wu, Y., Kirillov, A., Massa, F., Lo, W.-Y. & Girshick, R. Detectron2. https://github.com/facebookresearch/detectron2 (2019).

[20] Yun K, Park J, Cho J (2020). Robust human pose estimation for rotation via self-supervised learning. IEEE Access.

[21] Chen, Y. *et al.* Cascaded pyramid network for multi-person pose estimation. In *Proceedings of the IEEE Conference on Computer Vision and Pattern Recognition*, 7103–7112 (2018).

[22] Andriluka, M., Roth, S. & Schiele, B. Pictorial structures revisited: People detection and articulated pose estimation. In *2009 IEEE Conference on Computer Vision and Pattern Recognition*, 1014–1021 (IEEE, 2009).

[23] Dantone, M., Gall, J., Leistner, C. & Van Gool, L. Human pose estimation using body parts dependent joint regressors. In *Proceedings of the IEEE Conference on Computer Vision and Pattern Recognition*, 3041–3048 (2013).

[24] Gkioxari, G., Arbeláez, P., Bourdev, L. & Malik, J. Articulated pose estimation using discriminative armlet classifiers. In *Proceedings of the IEEE Conference on Computer Vision and Pattern Recognition*, 3342–3349 (2013).

[25] Johnson, S. & Everingham, M. Learning effective human pose estimation from inaccurate annotation. In *CVPR 2011*, 1465–1472 (IEEE, 2011).

[26] Pishchulin, L., Andriluka, M., Gehler, P. & Schiele, B. Poselet conditioned pictorial structures. In *Proceedings of the IEEE Conference on Computer Vision and Pattern Recognition*, 588–595 (2013).

[27] Sapp, B. & Taskar, B. Modec: Multimodal decomposable models for human pose estimation. In *Proceedings of the IEEE Conference on Computer Vision and Pattern Recognition*, 3674–3681 (2013).

[28] Yang, Y. & Ramanan, D. Articulated pose estimation with flexible mixtures-of-parts. In *CVPR 2011*, 1385–1392 (IEEE, 2011).

[29] Newell, A., Yang, K. & Deng, J. Stacked hourglass networks for human pose estimation. In *European Conference on Computer Vision*, 483–499 (Springer, 2016).

[30] Gkioxari, G., Toshev, A. & Jaitly, N. Chained predictions using convolutional neural networks. In *European Conference on Computer Vision*, 728–743 (Springer, 2016).

[31] Bulat, A. & Tzimiropoulos, G. Human pose estimation via convolutional part heatmap regression. In *European Conference on Computer Vision*, 717–732 (Springer, 2016).

[32] Insafutdinov, E., Pishchulin, L., Andres, B., Andriluka, M. & Schiele, B. Deepercut: A deeper, stronger, and faster multi-person pose estimation model. In *European Conference on Computer Vision*, 34–50 (Springer, 2016).

[33] Wei, S.-E., Ramakrishna, V., Kanade, T. & Sheikh, Y. Convolutional pose machines. In *Proceedings of the IEEE conference on Computer Vision and Pattern Recognition*, 4724–4732 (2016).

[34] Yang, W., Li, S., Ouyang, W., Li, H. & Wang, X. Learning feature pyramids for human pose estimation. In *Proceedings of the IEEE International Conference on Computer Vision*, 1281–1290 (2017).

[35] Papandreou, G. *et al.* Towards accurate multi-person pose estimation in the wild. In *Proceedings of the IEEE Conference on Computer Vision and Pattern Recognition*, 4903–4911 (2017).

[36] Huang, S., Gong, M. & Tao, D. A coarse-fine network for keypoint localization. In *Proceedings of the IEEE International Conference on Computer Vision*, 3028–3037 (2017).

[37] Newell, A., Huang, Z. & Deng, J. Associative Embedding: End-to-End Learning for Joint Detection and Grouping. *arXiv e-prints* arXiv:1611.05424 (2016). 1611.05424.

[38] Pishchulin, L. *et al.* Deepcut: Joint subset partition and labeling for multi person pose estimation. In *Proceedings of the IEEE Conference on Computer Vision and Pattern Recognition*, 4929–4937 (2016).

[39] Rhodin, H. *et al.* Learning monocular 3D human pose estimation from multi-view images. In *Proceedings of the IEEE Conference on Computer Vision and Pattern Recognition*, 8437–8446 (2018).

[40] Ostrek M, Rhodin H, Fua P, Müller E, Spörri J (2019). Are existing monocular computer vision-based 3D motion capture approaches ready for deployment? A methodological study on the example of alpine skiing. Sensors.

[41] Bachmann, R., Spörri, J., Fua, P. & Rhodin, H. *Motion capture from pan-tilt cameras with unknown orientation* vol. 1908, 11676 (2019).

[42] Spörri J (2016). Reasearch dedicated to sports injury prevention-the’sequence of prevention’on the example of alpine ski racing. Habilit. Venia Docendi Biomech.

[43] Barth M, Platzer HP, Giger A, Nachbauer W, Schröcksnadel P (2021). Acute on-snow severe injury events in elite alpine ski racing from 1997 to 2019: The injury surveillance system of the Austrian ski federation. Br. J. Sports Med..

[44] Dijkstra EW (1959). A note on two problems in connexion with graphs. Numerische mathematik.

[45] Bere T (2011). Mechanisms of anterior cruciate ligament injury in world cup alpine skiing: A systematic video analysis of 20 cases. Am. J. Sports Med..

